# Microbiota signatures relating to reduced memory and exploratory behaviour in the offspring of overweight mothers in a murine model

**DOI:** 10.1038/s41598-019-48090-8

**Published:** 2019-08-30

**Authors:** Elena Sanguinetti, Maria Angela Guzzardi, Maria Tripodi, Daniele Panetta, Marta Selma-Royo, Alessandro Zega, Mauro Telleschi, Maria Carmen Collado, Patricia Iozzo

**Affiliations:** 1 Institute of Clinical Physiology, National Research Council (CNR) via Moruzzi 1, 56124 Pisa, Italy; 2 Sant’Anna School of Advanced Studies, Pisa, Piazza Santa Caterina, 56100 Pisa, Italy; 3 Institute of Agrochemistry and Food Technology-National Research Council (IATA-CSIC), 46980 Valencia, Spain

**Keywords:** Obesity, Bacterial host response

## Abstract

An elevated number of women of reproductive age are overweight, predisposing their offspring to metabolic and neuropsychiatric disorders. Gut microbiota is influenced by maternal factors, and has been implicated in the pathogenesis of neurodegenerative diseases. Our aim was to explore the effects of maternal high-fat feeding on the relationship linking gut microbiota and cognitive development in the offspring. Murine offspring born to dams undergoing normal diet (NDm) and high-fat diet (HFDm) were studied at 1 or 6 months of age to assess cognitive function by Y-maze test, cerebral glucose metabolism and insulin sensitivity by Positron Emission Tomography, brain density by Computed Tomography, microbiota profile (colon, caecum) and inferred metabolic pathways (KEGG analysis) by 16S ribosomal RNA sequencing. From 3 weeks post-weaning, mice born to HFDm developed hyperphagia and overweight, showing reduction in memory and exploratory behaviour, and brain insulin resistance in adulthood. We identified a panel of bacteria characterizing offspring born to HFD dams from early life, and correlating with dysfunction in memory and exploratory behaviour in adults (including Proteobacteria phylum, *Parabacteroides* and unclassified *Rikenellaceae* genera). Microbiota-derived metabolic pathways involved in fatty acid, essential aminoacid and vitamin processing, sulphur metabolism, glutaminergic activation and Alzheimer’s disease were differently present in the HFDm and NDm offspring groups. Our results document tight relationships between gut dysbiosis and memory and behavioural impairment in relation to maternal HFD. Persistent bacterial signatures induced by maternal HFD during infancy can influence cognition during adulthood, opening the possibility of microbiota-targeted strategies to contrast cognitive decline.

## Introduction

The burden of cognitive dysfunction is increasing due to population aging, and to a growing prevalence of predisposing conditions, such as obesity^1^. Mental health disorders (either emotional, behavioural or other) have high lifetime prevalence, with first onset usually in childhood or adolescence^2^. Brain and cognitive development prevail in early life, predicting later brain health^3–5^. Thus, it may be possible and important to recognize and alleviate early risk determinants. Early life phases are characterized by gut microbiota colonization^6,7^. Gut bacteria regulate body metabolism, eating behaviour, adiposity and systemic inflammation^8,9^, and have been recently associated with cognitive disorders in adult humans^10^ and rodents^11,12^. In an adult mouse model of cognitive pathology, we have identified specific microbiota signatures, and additive effects of high-fat feeding^13^, correlating with cognitive dysfunction and brain glucose metabolism. We also observed that brain metabolic responses to acute intranasal insulin administration were compromised^14^, in line with the involvement of cerebral insulin resistance in neurodegenerative conditions^15,16^.

A growing number of pregnant women are overweight^17^, and gestational overweight associates with lower cognitive abilities and behavioural disorders in the offspring, including anxiety, depressive-like behaviour, autism spectrum disorders, attention deficit hyperactivity disorders^18,19^, from childhood^20–24^, but not yet at weaning^25,26^. In rodents, maternal high-fat diet (HFD) results in systemic and hippocampal-hypothalamic inflammation^18^ and damage^27–29^, with persistent changes in behaviour and learning^18^, food intake and energy expenditure^30^. Few studies have examined the effects of maternal obesity or HFD on the offspring’s gut microbiota. Some show reductions in *Bacteroides*^31^, Proteobacteria^32,33^, and *Eubacterium* and *Blautia*^34^, and increases in *Enterococcus*^31^, *Bacteroides* and *Staphylococcus*^35^, or *Oscillibacter*, *Parabacteroides* and unclassified Bacteroidales^34^. Others report very limited and short-lasting effects^36–38^. In rodents and primates, the impact of maternal HFD on gut microbiota composition was not fully normalized by post-natal control diet^39,40^.

In the current study, we hypothesized that brain function and gut microbiota are influenced by maternal gestational HFD, and that the gut microbiota might explain the dual dysregulation in eating behaviour and memory and exploratory behaviour in the offspring of HFD dams. We also considered that the bacterial taxa relating to brain function in early and later life might differ, and their distinct identification is important when considering microbiota-based preventive strategies along life-course phases. Our aim was to examine, in a murine model, the effects of maternal HFD on memory and exploratory behaviour, cerebral metabolism, gut microbiota, and their relationship at weaning and adult age, accounting for eating behaviour, body weight, and inflammatory profiles. Since the microbiota has different composition in the adult caecum and colon^13^, we examined both intestinal tracts.

## Results

### General characterization

**Figure 1:**
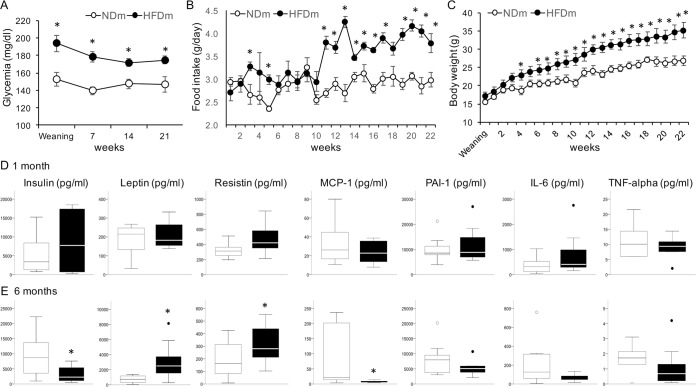
Glycemia, food intake, body weight and circulating markers. Offspring of HFD mothers showed higher random glycemia (**A**) and daily food intake (**B**) at weaning and throughout growth compared to offspring of ND mothers, consistent with a greater body weight starting from 5 weeks of age (**C**). Circulating levels of insulin and cytokines were not different between groups at weaning (**D**); at 6 months of age the offspring of HFD mothers showed higher leptin and resistin, and lower insulin and MCP1 levels compared to age-matched controls (**E**). In line graphs, data are presented as mean ± SEM. In boxplots, black points represent outliers, i.e. cases with values between 1.5 and 3 times the interquartile (IQ) range. *p < 0.05.

As expected, HFD mothers (HFDm) showed ∼28% heavier body weight (31.5 ± 3.9 vs 24.8 ± 0.5 g, p = 0.18) and ∼11% higher glycemia (8.3 ± 0.2 vs 7.5 ± 0.1 mmol/L, p = 0.004), consistent with greater energy intake from fat (956 ± 247 vs 103 ± 12 kcal, p = 0.012), compared to ND mothers (NDm). HFDm offspring were characterized by significantly higher random glycemia compared to controls (Fig. 1A). Daily food intake was significantly greater in mice born to HFD dams, starting 3 weeks post-weaning, and more markedly after 3 months of age (adulthood) (Fig. 1B). Consequently, overweight was progressive in this group, starting from 5 weeks post-weaning (Fig. 1C). At 6 months, HFDm offspring showed elevated leptin and resistin, and lower insulin and monocyte chemoattractant protein-1 (MCP-1) levels compared to age-matched controls (Fig. 1D,E).

### Memory, exploratory behaviour and cerebral metabolism

**Figure 2:**
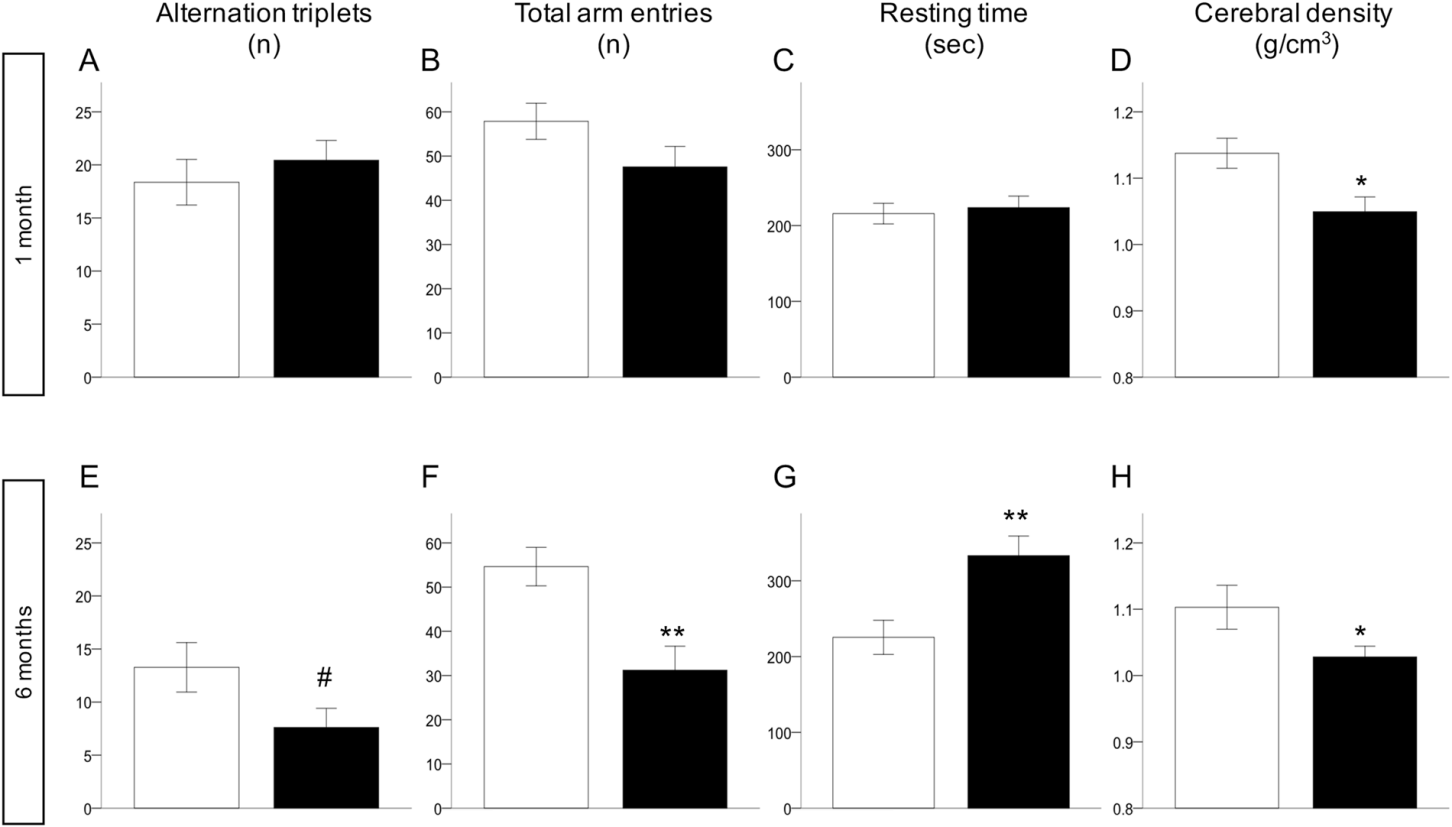
Cognitive function parameters, brain density at weaning (**A**–**D**) and 6 months (**E**–**H**). White bars (NDm), black bars (HFDm). Data are presented as mean ± SEM, **p < 0.01, *p < 0.05, ^#^p ≤ 0.07.

At weaning, memory (alternation triplets) and locomotor (total arm entries and distance travelled) parameters were not reduced in HFDm than NDm offspring (Fig. 2A–C). Latency and resting time, travelled distance and speed were not different between groups. In adult HFDm offspring (Fig. 2E–G), all Y-maze parameters were impaired compared to controls. HFDm offspring showed significantly lower cerebral density compared to age-matched controls (Fig. 2D,H). Leptin was correlated inversely with memory (alternation triplets) and exploratory function (total arm entries and distance travelled) at one month (r = −0.51–075, p < 0.05), and at 6 months (r = −0.49–0.61, p < 0.05).

**Figure 3:**
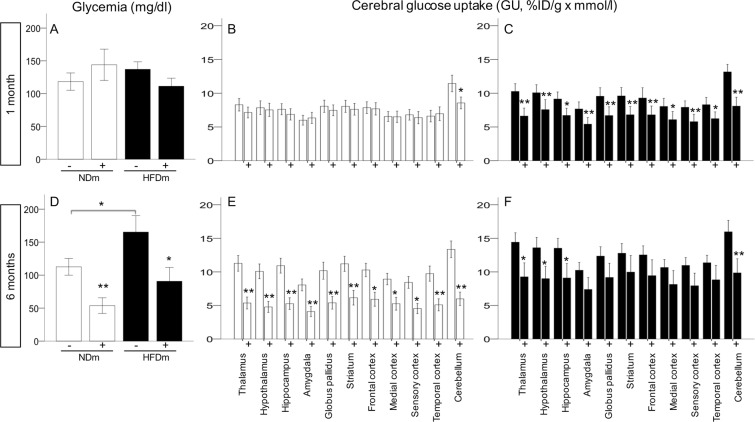
Systemic (**A**,**D**) and cerebral (**B**,**C**,**E**,**F**) effects of intranasal insulin administration. Circulating glucose levels and cerebral glucose uptake were measured in fasting conditions (−) and in response to acute intranasal insulin administration (+). Data are presented as mean ± SEM, **p < 0.01, *p < 0.05.

Intranasal insulin (INI) administration reduced systemic glucose levels in adults (Fig. 3A,D). At baseline, brain glucose uptake (GU) was not different between groups (Fig. 3B,C,E,F). In NDm offspring, intranasal insulin delivery caused a 40–50% decrease from baseline in brain glucose uptake during adulthood (Fig. 3E). Instead, HFDm offspring were characterized by a significant response to insulin during infancy (Fig. 3C) and a blunted response during adulthood (Fig. 3F).

### Microbiota composition in caecum and colon

**Figure 4:**
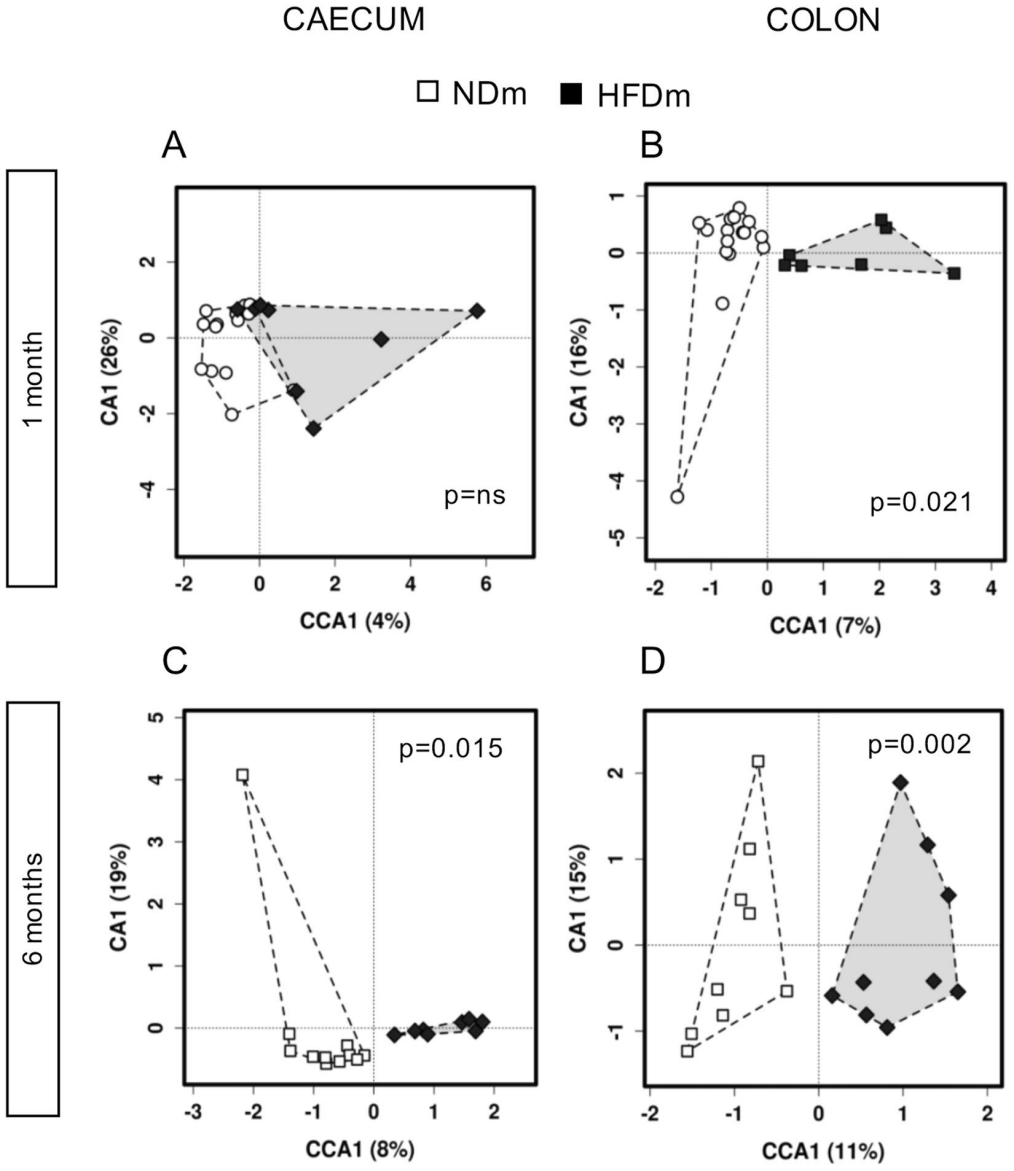
Multivariate canonical correspondence analysis (CCA) of gut microbiota. Association between maternal habitus (NDm vs HFDm) and microbiota composition (OTU level) at 1 month (**A**,**B**) and 6 months of age (**C**,**D**) in the caecum and colon. Microbiota composition was significantly separated between groups at both 1 and 6 months of age in the colon (**B**,**D**), and only at 6 months of age in the caecum (**C**).

**Figure 5:**
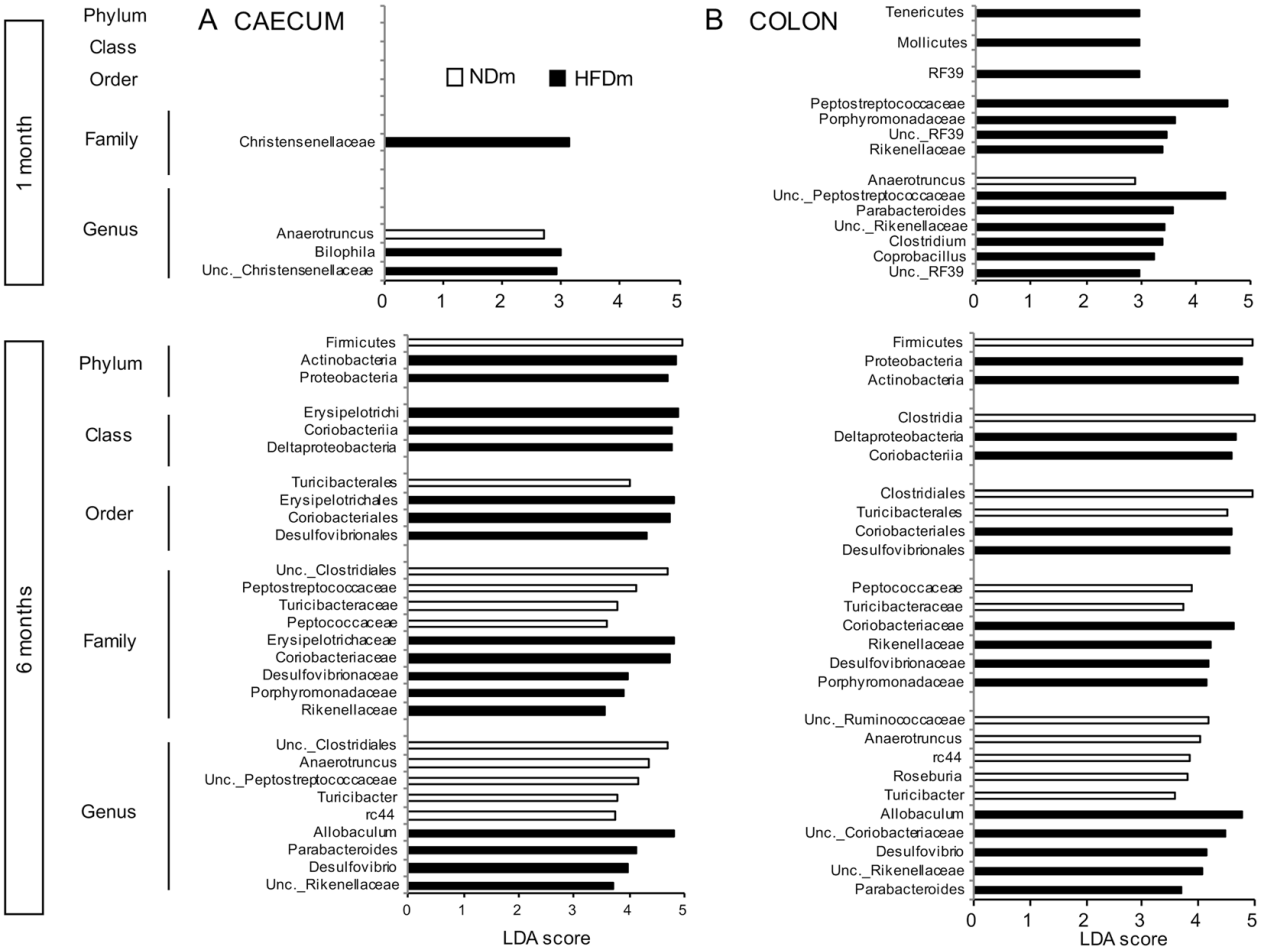
Linear discriminanant analysis (LDA) effect size method (LEfSE) of gut microbiota. Bacteria taxa at phylum, class, order, family and genus levels (LDA score >3) explaining the difference in microbiota composition in the caecum (**A**) and colon (**B**), between offspring of mothers fed normal diet (NDm) or high-fat diet (HFDm) at 1 and 6 months of age.

Based on canonical correspondence analysis (CCA) of microbiota composition (Table S1, Fig. 4), group differences occurred earlier in the colon than in the caecum. Microbiota composition (e.g. operational taxonomic units, OTUs, Fig. 4) was clearly separated between NDm and HFDm offspring at both 1 and 6 months of age in the colon (Fig. 4B,D), and only at 6 months of age in the caecum (Fig. 4C). These findings were consistent across taxa levels (phylum, order, family, genus) (Table S1). In 1 month old mice, no enrichment or depletion in the microbiota profile was observed at phylum, class and order level in the caecum (Fig. 5A) in HFDm compared to NDm; in the colon, the Tenericutes phylum, Mollicutes class, and RF39 order showed significant predominance in HFDm offspring (Fig. 5B). More group-specific microbiota hallmarks were observed at family, genus (Fig. 5B) and OTUs levels (Fig. S1A,B). At 6 months, there were significant discriminant biomarkers at all taxa levels. Both in colon and caecum, Proteobacteria and Actinobacteria phyla prevailed in 6 months old HFDm offspring, whereas Firmicutes prevailed in 6 months old NDm offspring.

### Associations between colon microbiota and memory and exploratory behaviour

**Figure 6:**
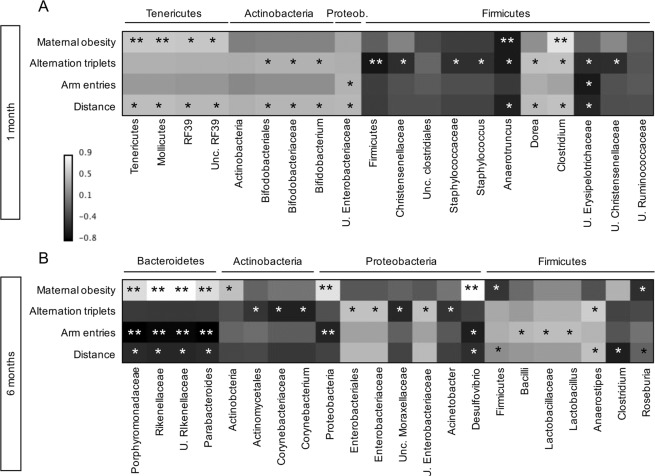
Correlation between maternal habitus, memory and behavioural parameters and colon microbiota. Heatmaps showing Spearman’s correlation coefficients linking colon microbiota taxa and maternal obesity (ND vs HFD), memory and exploratory behaviour parameters at 1 month (**A**) and 6 months of age (**B**). Assigned phyum is indicated above bacterial names. *p ≤ 0.05 in the univariate analysis, **p ≤ 0.05 in the FDR-corrected analysis.

In one month old mice (Fig. 6A), a strong correlation was observed between the increase in Firmicutes phylum bacteria and the decline in alternation triplets (reflecting memory and cognitive function), remaining significant after controlling false discovery rate (FDR) by Benjamini-Hochberg procedure. Bacteria explaining this negative correlation belonged to Bacilli (Staphylococcaceae family, *Staphylococcus* genus), Clostridia (Christenellaceae and Clostridiaceae families), and Erysipelotrichia classes (Erysipelotrichaceae family). Positive correlations were observed between cognitive function and the *Dorea* genus (Firmicutes phylum), *Bifidobacterium* genus (Actinobacteria phylum), and unclassified Enterobacteriaceae genera (Proteobacteria phylum). The only genera that were associated with both maternal habitus and cognitive function were the *Aanerotruncus* and *Clostridium* genera (Clostridia class).

In 6 months old mice (Fig. 6B), a strong correlation was observed between the increase in Proteobacteria phylum bacteria and the decline in total arm entries, reflecting exploratory behaviour, remaining significant after FDR adjustment. Abundance in genera belonging to Proteobacteria phylum (unclassified *Moraxellaceae*, *Acinetobacter*), and Actinobacteria phylum (*Corynebacterium*) were predictive of lower cognition, whereas higher cognition was related with an increased abundance of *Anaerostipes* (Firmicutes phylum) and unclassified Enterobacteriaceae (Proteobacteria phylum). Exploratory behaviour was strongly associated (surviving FDR correction) with reduction in the abundance of Proteobacteria phylum (mainly in *Desulfovibrio spp*.), and Porphyromonadaceae, *Parabacteroides*, unclassified Rikenellaceae (Bacteroidetes phylum), while showing positive relationships with relative abundance of *Lactobacillus* (Firmicutes phylum). Notably, the panel showing strongest negative correlations with memory or exploratory behavior, i.e. surviving FDR adjustment (1 phylum: Proteobacteria, p = 0.049, 2 families: Rikenellaceae and Porphyromonadaceae, p = 0.001, and 2 genera: *Parabacteroides* and unclassified *Rikenellaceae*, p = 0.002, with *Desulfovibrio*, *p* = *0*.*056*), was also identified in LEfSe analysis in 6 months old HFDm offspring (Fig. 5B), and most of its components (Rikenellaceae and Porphyromonadaceae families, and unclassified Rikenellaceae and Parabacteroides genera) were already increased in HFDm offspring at the age of 1 month (Fig. 5A). In addition, among these genera, *Desulfovibrio*, *Parabacteroides* and unclassified Rikenellaceae were also positively correlated with food intake or body weight in adults, though only in univariate analysis (Table S2).

### Microbiota metabolic pathways (KEGG) in colon

**Figure 7:**
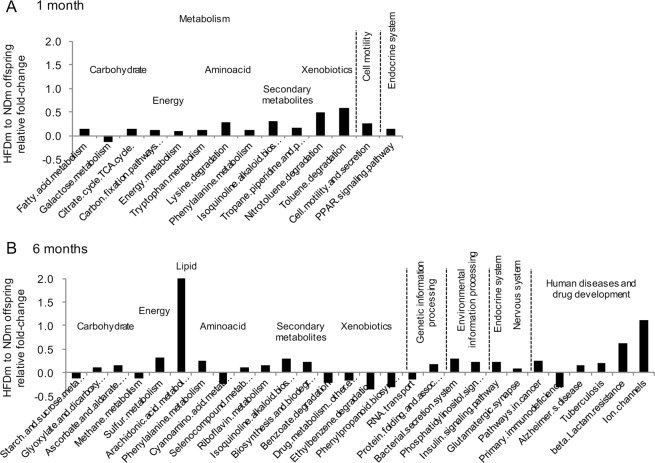
Colon microbiota functional analysis. Predictive functional analysis using PICRUSt with Kyoto Encyclopaedia of Genes and Genomes (KEGG) revealed colon microbiota-derived pathways that were differently regulated in the offspring born to mothers fed high-fat versus normal diet, both at 1 (**A**) and 6 months of age (**B**). The identified pathways are implicated in metabolism, genetic information processing, environmental information processing, cell motility, endocrine and nervous system and human diseases, and drug development. Pathways with a relative fold-change >10% and a significant difference (p < 0.05) between NDm and HFDm offspring in the Wilcoxon-rank test are shown.

In global multivariate analyses of KEGG derived metabolic pathways, a significant difference was found in the colon (p = 0.05 by CCA) in 6 months old NDm and HFDm offspring (Table S3). No global difference was observed in the caecum or in 1 month old animals. At the level of specific KEGG derived pathways, fold-change and LEfSe analyses (Figs 7A,B, S2) revealed significant group differences at both ages, with HFDm offspring showing upregulation of pathways involved in energy metabolism, (mostly essential) aminoacids (phenylalanine at 1 and 6 months, lysine and tryptophan at 1 month, cianoaminocid at 6 months), glucose and lipid metabolism, secondary metabolites, and endocrine system. At 6 months, there was also overexpression of pathways involved in sulfur metabolism and nervous system, i.e. glutamatergic synapse, protein folding, phosphatydilinositol signaling, arachidonic acid, ion channels and Alzheimer’s disease.

## Discussion

The main novelties of this study consist in the identification of early gut signatures that persist through adulthood, which depend on maternal diet and strongly predict adulthood cognitive and exploratory behaviour, and in the quantification of brain responses to intranasal insulin stimulation *in vivo*, implicating cerebral insulin resistance in the cognitive deficit induced by maternal HFD.

Cognitive diseases are increasing in prevalence worldwide. Prenatal and early postnatal experiences are fundamental in brain development, and determine cognitive risk in adulthood^3–5^. Maternal pregravid body mass index (BMI) negatively predicts preschool and school children cognition^21,22,24^ and development of other neuropsychiatric disorders such as anxiety, depressive-like behaviour, autism spectrum disorders, attention deficit hyperactivity disorders^19^, but there seems to be a time-window, from birth to weaning^23,25,26^, in which the negative effect of maternal obesity is not yet clinically detectable. In keeping with human data, we observed that memory and locomotor parameters were normal in HFDm offspring at end-lactation compared to age-matched controls. Conversely, a marked impairment in both functions was seen in adults, consistent with previous reports^18,28^. The earliest brain abnormality was a reduction in cerebral density, which is coherent with the impairment in neurogenesis^18,27,41^ seen in other offspring studies, and with cerebral density losses typically seen in patients with cognitive dysfunction and Alzheimer’s disease^42–44^. Our HFDm pups had normal weight and fasting glucose levels, consistent with previous evidence in mice^39^, though random glucose assessments were suggestive of post-prandial hyperglycemia, likely due to fat consumption in breast milk. HFDm offspring became hyperphagic after 3 weeks post-weaning, consistent with the early programming of circuitries involved in appetite control^45–47^. They progressively showed overweight, hyperglycemia, -leptinemia, -resistinemia and low insulin and MCP-1 levels compared to NDm offspring. Impaired beta cell insulin secretion in adult offspring of obese mothers has been previously reported^48,49^, although the mechanism needs to be better explored. MCP-1 expression is insulin-responsive^50^, thus the decreased insulin secretion found in adult offspring of HFD mother in our model might be responsible for lower MCP-1. Overall, these hormonal changes support the link of memory impairment, peripheral dysmetabolism and central insulin resistance^51^.

Cerebral insulin resistance has been implicated in the pathogenesis of Alzheimer’s disease^15,16^, and our findings extend this association to the offspring of HFD mothers. In the present study, we examined brain GU before and after a direct administration of insulin in the brain, without manipulating peripheral glycemia. Intranasal insulin administration resulted in a lowering of glycemia, reducing brain GU in adult controls, which is coherent with previous studies showing that cerebral insulin delivery stimulates hepatic glucose uptake and conversion into glycogen^52–54^, and reduced endogenous glucose production (i.e. liver glucose production)^55,56^. In our model, brain response to INI in the offspring of HFDm was significantly greater at weaning and blunted (statistical significance reached only in 4 brain regions out of 11 examined) in adult age compared to controls. This is consistent with our previously published data showing that during peripheral insulin infusion in a hyperinsulinaemic-isoglycaemic clamp, brain glucose uptake in minipigs of HFD mothers was elevated in early life, and defective during young adulthood, being paralleled by cortical and hypothalamic expression of insulin receptors and insulin-sensitive glucose transporters^57^. The study suggested that the brain of animals born to obese sows was initially hypersensitive to insulin, and then became insulin resistant^57^. However, we could not measure cognitive function in pigs, and insulin was not selectively administered into the brain. In the present study, we demonstrate that brain insulin sensitivity was present together with normal memory and explorative performance in early HFDm offspring, whereas cerebral insulin resistance occurred together with impairment in memory and explorative behaviour in adults born to HFD mothers. These timelines might suggest that maternal obesity associates with early brain maturation followed by early deterioration in the offspring, and that there might be a time-window for effective prevention.

In recent years, intensive research efforts have been devoted to the identification of microbiota-based targets for early risk detection and prevention of chronic diseases, and strong support of a causal link between microbiota composition and neurodegeneration has been provided^58^. Therefore, we focused on the identification of bacterial candidates that may be linked to cognitive or behavioural disorders and its impairment in the offspring of HFD dams. Our results confirm the excess observed in Firmicutes in early life in offspring of HFD dams^39^, but the most relevant finding was that 1 and 6 months old HFDm offspring showed the same dominance of Parabacteriodes (Porphyromonadaceae) and unclassified Rikenellaceae (Bacteroidetes phylum) observed in strong, i.e. FDR adjusted correlation with impaired exploratory behviour in adults. *Desulfovibrio* genus was also inversely associated with exploratory behviour in adult mice, falling short of significance in FDR adjustment (p = 0.056). Interestingly, an increase in *Parabacteroidetes* has been described also in children born to obese mothers, conferring translational potential to our results^34^. The identified taxa are coherent with observations in cognitively impaired SAMP8 mice, showing greater abundance of Proteobacteria-Desulfovibrionaceae and Rikenellaceae, and low abundance of *Ruminococcus* and *Anaerotruncus* in coherent association with cognitive abilities^59^. Notably, these predictors of impairment in memory or exploratory function were not independently associated with food intake and/or overweight, after FDR correction. In synthesis, the above findings suggest that adverse microbiota changes induced by maternal HFD and associating with impairment in memory and exploratory function are seeded before weaning, and manifest their unfavourable cerebral action between weaning and adulthood.

We found also bacterial taxa that correlated with brain functional features, but were not dependent on maternal diet. The ones correlating with low cognition mostly belonged to the Firmicutes phylum in early life, and to Corynebacteriaceae (*Corynebacterium*) and Moraxellaceae (*Acinetobacter*) families in adults, whereas Enterobacteriaceae (Proteobacteria phylum) and Bifidobacteriaceae families (*Bifidobacterium* genus, Actinobacteria phylum) in early life, and *Lactobacillus*, *Anaerostipes* and Unclassified Enterobacteriaceae genera in adults were related to better memory and greater exploratory activity, and may be protective. Though most of these correlations lost full significance after FDR adjustment, they are in line with our reported dominance (Firmicutes phylum, Christenellaceae family, *Staphylococcus* genus) or depletion of specific bacteria taxa (Bifidobacteriaceae family, *Bifidobacterium* genus) in adult mice with genetically induced Alzheimer’s type pathology^13^, and with the evidence that *Lactobacillus*- and *Bifidobacterium*-based probiotics ameliorate minimental state examination score (+27%) and insulin sensitivity (+28%) in patients with Alzheimer’s disease in 12 weeks^60^.

Different mechanisms have been proposed to explain the cross-talk between gut bacteria and the central nervous system of mammals, including direct diffusion of microbiota metabolites across the gut wall, or the transport of microbiota-derived neurotransmitters via afferent neural circuits^61^. We used KEGG pathway analysis to translate our microbiota composition data into biologically meaningful pathways, and most of the emerging pathways are recognized regulators of neurological function and appetite control. We found that lysine, phenylalanine, tryptophan pathways were overexpressed in HFDm offspring. Though essential aminoacids are required for health, their excess impairs neurological development^62^. Lysine is involved in epigenetic regulation^63^, and this pathway was affected in early life, when epigenetic modifications are prominent; moreover, the consensual change in fatty acid metabolic, TCA cycle, cell motility and secretion pathways at this age was in line with the known involvement of this aminoacid in regulating calcium intestinal absorption^64^, and fatty acid mitochondria transport and oxidation^65^. Among appetite regulators, tryptophan at 1 month and arachidonic acid at 6 months, were identified. The former is precursor of serotonin, and mostly produced by the gut to act on the brain and regulate appetite^66^. Arachidonic acid was elevated by 2 folds in HFDm compared to NDm offspring, representing the highest group difference among all pathways. Arachidonic acid is precursor of the orexogenic endocannabinoids anandamide^67^, and the most abundant fatty acid in the brain, with a fundamental role in early neurological development and neurodegenerative disease^68^. Other disturbances in pathways related to neurodevelopment and neurodegeneration in our HFDm offspring were the overexpression of the phenylanine (precursor of tyrosine and L-DOPA^69^) pathway, underexpression of the cyano-aminoacid (precursor of asparagine, essential for glutamate formation) pathway, and upregulation of the glutamatergic-synapse and ion channels pathways (favoring the dominance of excitatory versus inhibitory functions), as typically observed in neurodegenerative disorders^70^. Several gut bacteria are able to produce glutamate or GABA^71^, and it is of note that *Corynebacterium*, which is the main gut producer of glutamine, was a negative predictor, whereas *Lactobacillus* and *Bifidobacterium*, known to metabolize glutamate and produce GABA^71^, were positive predictors of cognitive function. The upregulation of the sulfur metabolic pathway is also coherent with the excess in aminoacids (source of sulfur) and the high abundance of *Desulfovibrio* observed in HFDm offspring. One important observation was that, 6 months old HFD offspring displayed a significant upregulation of the Alzheimer’s disease pathway, covering a large series of strictly pathogenetic genetic changes. Overall, remodeling of microbiota composition resulted in a metabolic configuration that could explain overeating behavior and impairment in memory and exploratory behavior in the offspring of HFD mothers.

Our study conveys novelty, but has limitations. To our knowledge, our results are the first to show the relationship linking brain dysfunction and dysbiosis, as a result of short- and long-term effects of maternal HFD, and to extend the evidence of cerebral insulin resistance to HFDm offspring, by using *in vivo* imaging approaches. However, our correlative analyses are not sufficient to dissect the causal role of maternal obesity versus microbiota changes (induced by maternal obesity) on the phenotype observed in the offspring. We also recognize that additional cognitive and behavioural tests or microbiota transplantation experiments, and molecular measurements may have added further support and insight (in)to our findings. A technical limitation in cerebral ^18^F-2-fluoro-2-deoxyglucose-positron emission tomography (FDG-PET) studies is that anaesthesia may lead to systematic underestimation in brain metabolism, though its influence on group comparisons was minimized by strictly reproducing the same protocol in all studies.

## Conclusions

In conclusion, our animal model demonstrates that overeating, memory decline, exploratory abnormalities and cerebral insulin resistance are delayed phenomena in the offspring of HFD dams, which leaves a useful time-window open to prevention maneuvers. We identified a well-defined panel of bacteria taxa that are dependent on maternal status and strongly related with behavioural dysfunction in adult mice, and we documented that these same bacteria are overrepresented since early life in the offspring of HFD dams. These findings open the possibility of microbiota-targeted strategies to contrast cognitive and behavioural deviations.

## Methods

### Study design

B6129SF2/J (stock no: 101045, The Jackson Laboratory, Bar Harbor, Maine) female mice were randomly divided in: 1) normal diet-fed mothers (NDm, 11% kcals from fat, Mucedola, Italy, n = 5), and 2) high-fat diet-fed mothers (HFDm, 58% kcals from fat, Mucedola, n = 4). Following 3 months of diet, females were mated (B6129SF2/J males), and allowed to deliver spontaneously. Maternal diets were maintained through gestation and lactation. After weaning, offspring (total n = 45, NDm = 27 and HFDm = 18) were fed with standard diet. Animals were housed under standard conditions (22 °C, 12-hour light/dark cycles), with *ad libitum* access to food and water. From weaning, offspring’s food intake and body weight were monitored weekly and glycemia every ∼7 weeks. At 1 (weaning, NDm/HFDm = 27/18 cognition and NDm/HFDm = 11/8 cerebral glucose metabolism) and 6 months of age (adulthood, NDm/HFDm = 11/10 cognition and NDm/HFDm = 10/8 cerebral glucose metabolism), cognitive function and cerebral glucose metabolism (at baseline and after intranasal insulin administration) were assessed. Then, animals were euthanized by anaesthetic overdose, and blood and brains collected. The experimental protocol was conducted in accordance with the D.L.116/92 implementation of EEC directive 609/86 regarding the protection of animals used for experimental and other scientific purposes. Veterinary and animal care staff was responsible for animal welfare and supervision.

### Y-maze test

Each animal was allowed to move freely through a standard 3-arm Y-maze (Panlab, Harvard Apparatus, Spain) for 8 minutes, under visual automatic tracking of latency time (until first arm choice), spontaneous alternation triplets (number of 3 consecutive entries in different arms), percentage of alternation triplets (against the maximum possible number), zone transition number, total arm entries, resting time, travelled distance and speed.

### Brain glucose metabolism

Imaging of ^18^F-2-fluoro-2-deoxyglucose by positron emission, computed tomography ([^18^F]FDG PET-CT, IRIS, Inviscan SAS, France) was performed under fasting and insulin-stimulated conditions. Anaesthesia was induced and maintained in overnight-fasted mice by ∼3% and ∼2% (v/v) isofluorane inhalation (IsoFlo®, Abbott Laboratories, IL, USA). Breath frequency and temperature were monitored during the study and a heated pad was used to prevent hypothermia. An intraperitoneal catheter was placed for tracer administration. A 60-minute whole-body dynamic PET scan was acquired in fasting conditions after [^18^F]FDG injection. Then, intranasal insulin was administered (0.29/0.87 UI in 8/24 μl, 1/6 months, Sigma-Aldrich, MO, USA), as described^20^. Thirty minutes later, [^18^F]FDG injection and PET scanning were repeated. Every 10 minutes, glycemia was measured in tail blood by glucometer (OneTouch, Johnson&Johnson Medical SpA, Italy). PET data were corrected for dead time and radioactive decay, reconstructed by three-dimensional iterative ordered-subset expectation-maximization (3D-OSEM) algorithm, and co-registered to CT images by AMIDE Medical Image Data Examiner 1.0.5. Regions of interest were manually drawn on PET images in correspondence of thalamus, hypothalamus, hippocampus, amygdala, globus pallidus, striatum, frontal, medial, sensory and temporal cortex, and cerebellum. Activity levels in the brain regions were divided by the injected dose per gram of body weight (%ID/g), representing regional glucose fractional extraction, and multiplied by glycaemia, to reflect regional glucose uptake rates^57^.

At the end of *in vivo* procedures, brains were collected and weighted in a subset of mice to determine their mass. Cerebral volumes were measured on computed tomography (CT) images by a semi-automatic segmentation procedure using the Seg3D software (v2.2.1). Cerebral density was computed as ratio of whole brain mass-to-volume.

### Biochemical analyses

Blood samples were collected at the end of imaging procedures. Interleukin-6 (IL-6), Tumor Necrosis Factor-α (TNF-α), resistin, Plasminogen Activator Inhibitor-1 (PAI-1), Monocyte Chemoattractant Protein-1 (MCP-1), leptin, and insulin were assessed in plasma by Luminex® xMAP® technology (Mouse Adipokine Magnetic Bead Panel, Merck-Millipore Corp., MO, USA), according to manufacturer’s instructions.

### Gut bacteria 16S rDNA gene sequencing

Total DNA was isolated from caecum and colon content pellets by MasterPure Complete DNA&RNA Purification Kit (Epicentre, Illumina, WI, USA), as previously described^72^. DNA was measured using a Qubit® 2.0 Fluorometer (Life Technology, CA, USA) and normalized to 10 ng/μL. The V3-V4 region of 16S rDNA gene was amplified by PCR using Illumina adapter overhang nucleotide sequences according to Illumina protocols. The multiplexing step was performed using Nextera XT Index Kit (Illumina, CA, USA). A Bioanalyzer DNA 1000 chip (Agilent Technologies, CA, USA) was used to check the PCR product, and libraries were sequenced using a 2 × 300 pb paired-end run (MiSeq Reagent kit v3) on a MiSeq-Illumina platform (FISABIO sequencing service, Valencia, Spain) according to manufacturer’s instructions (Illumina). For quality control, reagents employed for DNA extraction and PCR amplification were also sequenced.Quality assessment was performed by prinseq-lite program (min_length:50; trim_qual_right:20; trim_qual_type:mean; trim_qual_window:20^73^). R1 and R2 from sequencing where joined using fastq-join from ea-tools suite. Data were obtained using an ad-hoc pipeline written in R Statistics environment and data processing was performed by QIIME pipeline (version 1.9.0)^74^, as previously described^13^. Chimeric sequences and sequences that could not be aligned were removed. The clustered sequences were utilized to construct OTUs tables (97% identity), then classified into phylum, family, genus taxonomic levels based on the Greengenes database. Sequences not taxonomically classified, or belonging to Cyanobacteria and Chloroplasts (representing ingested plant material), were removed.

### Statistical analysis

Calypso software version 8.84 was used with total sum normalization for the statistical analysis of microbiota data. Multivariate test canonical correspondence analysis (CCA) was used to assess complex association between gut bacteria community composition in the offspring and maternal diet. Linear discriminant analysis (LDA) effect size (LEfSe), were used to identify unique biomarkers (LDA score >3.0) in relative abundance of bacterial taxonomy^75^. Spearman’s correlation analysis was used to explore univariate associations between bacteria taxa relative abundances and cognitive or anthropometric parameters. Predictive inferred functional analysis was performed using PICRUSt with Kyoto Encyclopedia of Genes and Genomes (KEGG)^76^. Then, CCA, LEfSe analysis and Wilcoxon-rank test were used to explore associations between KEGG pathways and maternal groups. FDR-correction was applied in multiple comparisons analyses.

SPSS for MAC (version 22, Chicago, IL, USA) was used for the statistical analysis of other data. Variables distribution was tested by Shapiro-Wilk test. For normally distributed variables (e.g. glycemia, anthropometric and cognitive parameters) and variables normalized by logarithmic transformation (i.e. PET data), comparisons between groups were performed by two-tailed T-test for independent and paired samples. Mann-Whitney U test was used when normal distribution was not achieved (i.e. food intake and circulating biochemical markers). In group comparisons, values >3 times the IQ range were excluded: 1 value for circulating leptin, PAI-1, resistin and TNF-alpha at weaning, 2 values for leptin and MCP-1 and 1 value for resistin and TNF-alpha at 6 months Results were presented as mean ± sem or median and interquartile range (circulating markers), P-values ≤ 0.05 were regarded as statistically significant.

### Ethics approval

The experimental protocol was conducted in accordance with the D.L.116/92 implementation of EEC directive 609/86 regarding the protection of animals used for experimental and other scientific purposes. Veterinary and animal care staff was responsible for animal welfare and supervision.

## Supplementary information

Supplementary information

## Data Availability

The datasets used and/or analysed during the current study are available from the corresponding author on reasonable request.

## References

[1] Alford S, Patel D, Perakakis N, Mantzoros CS (2018). Obesity as a risk factor for Alzheimer’s disease: weighing the evidence. Obes Rev.

[2] Kessler RC (2005). Lifetime prevalence and age-of-onset distributions of DSM-IV disorders in the National Comorbidity Survey Replication. Arch Gen Psychiatry.

[3] Mento G, Bisiacchi PS (2012). Neurocognitive development in preterm infants: insights from different approaches. Neurosci Biobehav Rev.

[4] Whalley LJ (2000). Childhood mental ability and dementia. Neurology.

[5] Gow AJ (2008). Mental ability in childhood and cognitive aging. Gerontology.

[6] Nagpal R (2016). Sensitive Quantitative Analysis of the Meconium Bacterial Microbiota in Healthy Term Infants Born Vaginally or by Cesarean Section. Front Microbiol.

[7] Collado MC, Rautava S, Aakko J, Isolauri E, Salminen S (2016). Human gut colonisation may be initiated in utero by distinct microbial communities in the placenta and amniotic fluid. Sci Rep.

[8] Dinan TG, Cryan JF (2017). Microbes, Immunity, and Behavior: Psychoneuroimmunology Meets the Microbiome. Neuropsychopharmacology.

[9] El Aidy S, Stilling R, Dinan TG, Cryan JF (2016). Microbiome to Brain: Unravelling the Multidirectional Axes of Communication. Adv Exp Med Biol.

[10] Vogt NM (2017). Gut microbiome alterations in Alzheimer’s disease. Sci Rep.

[11] Bauerl C, Collado MC, Diaz Cuevas A, Vina J, Perez Martinez G (2018). Shifts in gut microbiota composition in an APP/PSS1 transgenic mouse model of Alzheimer’s disease during lifespan. Lett Appl Microbiol.

[12] Zhang L (2017). Altered Gut Microbiota in a Mouse Model of Alzheimer’s Disease. J Alzheimers Dis.

[13] Sanguinetti E (2018). Microbiome-metabolome signatures in mice genetically prone to develop dementia, fed a normal or fatty diet. Sci Rep.

[14] Sanguinetti E (2019). Combined Effect of Fatty Diet and Cognitive Decline on Brain Metabolism, Food Intake, Body Weight, and Counteraction by Intranasal Insulin Therapy in 3xTg Mice. Front Cell Neurosci.

[15] Plum L, Schubert M, Bruning JC (2005). The role of insulin receptor signaling in the brain. Trends Endocrinol Metab.

[16] Liu CC (2015). Neuronal LRP1 regulates glucose metabolism and insulin signaling in the brain. J Neurosci.

[17] Kim SY (2010). Percentage of gestational diabetes mellitus attributable to overweight and obesity. Am J Public Health.

[18] Bilbo SD, Tsang V (2010). Enduring consequences of maternal obesity for brain inflammation and behavior of offspring. FASEB J.

[19] Rivera HM, Christiansen KJ, Sullivan EL (2015). The role of maternal obesity in the risk of neuropsychiatric disorders. Front Neurosci.

[20] Brion MJ (2011). Intrauterine effects of maternal prepregnancy overweight on child cognition and behavior in 2 cohorts. Pediatrics.

[21] Basatemur E (2013). Maternal prepregnancy BMI and child cognition: a longitudinal cohort study. Pediatrics.

[22] Rodriguez A (2010). Maternal pre-pregnancy obesity and risk for inattention and negative emotionality in children. J Child Psychol Psychiatry.

[23] Casas M (2013). Maternal pre-pregnancy overweight and obesity, and child neuropsychological development: two Southern European birth cohort studies. Int J Epidemiol.

[24] Tanda R, Salsberry PJ, Reagan PB, Fang MZ (2013). The impact of prepregnancy obesity on children’s cognitive test scores. Matern Child Health J.

[25] Torres-Espinola FJ (2015). Maternal Obesity, Overweight and Gestational Diabetes Affect the Offspring Neurodevelopment at 6 and 18 Months of Age–A Follow Up from the PREOBE Cohort. PLoS One.

[26] Hinkle SN (2012). Associations between maternal prepregnancy body mass index and child neurodevelopment at 2 years of age. Int J Obes (Lond).

[27] Niculescu MD, Lupu DS (2009). High fat diet-induced maternal obesity alters fetal hippocampal development. Int J Dev Neurosci.

[28] Page KC, Jones EK, Anday EK (2014). Maternal and postweaning high-fat diets disturb hippocampal gene expression, learning, and memory function. Am J Physiol Regul Integr Comp Physiol.

[29] Lepinay AL (2015). Perinatal high-fat diet increases hippocampal vulnerability to the adverse effects of subsequent high-fat feeding. Psychoneuroendocrinology.

[30] Velloso LA (2012). Maternal consumption of high-fat diet disturbs hypothalamic neuronal function in the offspring: implications for the genesis of obesity. Endocrinology.

[31] Chu DM (2016). The early infant gut microbiome varies in association with a maternal high-fat diet. Genome Med.

[32] Mueller NT (2016). Birth mode-dependent association between pre-pregnancy maternal weight status and the neonatal intestinal microbiome. Sci Rep.

[33] Lemas DJ (2016). Alterations in human milk leptin and insulin are associated with early changes in the infant intestinal microbiome. Am J Clin Nutr.

[34] Galley JD, Bailey M, Kamp Dush C, Schoppe-Sullivan S, Christian LM (2014). Maternal obesity is associated with alterations in the gut microbiome in toddlers. PLoS One.

[35] Collado MC, Isolauri E, Laitinen K, Salminen S (2010). Effect of mother’s weight on infant’s microbiota acquisition, composition, and activity during early infancy: a prospective follow-up study initiated in early pregnancy. Am J Clin Nutr.

[36] Stanislawski MA (2017). Pre-pregnancy weight, gestational weight gain, and the gut microbiota of mothers and their infants. Microbiome.

[37] Laursen, M. F. *et al*. Infant Gut Microbiota Development Is Driven by Transition to Family Foods Independent of Maternal Obesity. *mSphere* **1**, 10.1128/mSphere.00069-15 (2016). 10.1128/mSphere.00069-15 PMC4863607 27303699

[38] Bergstrom A (2014). Establishment of intestinal microbiota during early life: a longitudinal, explorative study of a large cohort of Danish infants. Appl Environ Microbiol.

[39] Myles IA (2013). Parental dietary fat intake alters offspring microbiome and immunity. J Immunol.

[40] Ma J (2014). High-fat maternal diet during pregnancy persistently alters the offspring microbiome in a primate model. Nat Commun.

[41] Chen H, Simar D, Morris MJ (2014). Maternal obesity impairs brain glucose metabolism and neural response to hyperglycemia in male rat offspring. J Neurochem.

[42] Dos Santos V (2011). Morphological cerebral correlates of CERAD test performance in mild cognitive impairment and Alzheimer’s disease. J Alzheimers Dis.

[43] Makizako H (2013). Poor balance and lower gray matter volume predict falls in older adults with mild cognitive impairment. BMC Neurol.

[44] Schmidt-Wilcke T, Poljansky S, Hierlmeier S, Hausner J, Ibach B (2009). Memory performance correlates with gray matter density in the ento-/perirhinal cortex and posterior hippocampus in patients with mild cognitive impairment and healthy controls–a voxel based morphometry study. Neuroimage.

[45] Nivoit P (2009). Established diet-induced obesity in female rats leads to offspring hyperphagia, adiposity and insulin resistance. Diabetologia.

[46] Samuelsson AM (2008). Diet-induced obesity in female mice leads to offspring hyperphagia, adiposity, hypertension, and insulin resistance: a novel murine model of developmental programming. Hypertension.

[47] Chang GQ, Gaysinskaya V, Karatayev O, Leibowitz SF (2008). Maternal high-fat diet and fetal programming: increased proliferation of hypothalamic peptide-producing neurons that increase risk for overeating and obesity. J Neurosci.

[48] Han J, Xu J, Epstein PN, Liu YQ (2005). Long-term effect of maternal obesity on pancreatic beta cells of offspring: reduced beta cell adaptation to high glucose and high-fat diet challenges in adult female mouse offspring. Diabetologia.

[49] Zambrano E (2016). Decreased basal insulin secretion from pancreatic islets of pups in a rat model of maternal obesity. J Endocrinol.

[50] Sartipy P, Loskutoff DJ (2003). Monocyte chemoattractant protein 1 in obesity and insulin resistance. Proc Natl Acad Sci USA.

[51] Benomar Y (2013). Central resistin overexposure induces insulin resistance through Toll-like receptor 4. Diabetes.

[52] Pocai A (2005). Hypothalamic K(ATP) channels control hepatic glucose production. Nature.

[53] Ramnanan CJ (2011). Brain insulin action augments hepatic glycogen synthesis without suppressing glucose production or gluconeogenesis in dogs. J Clin Invest.

[54] Lam TK, Gutierrez-Juarez R, Pocai A, Rossetti L (2005). Regulation of blood glucose by hypothalamic pyruvate metabolism. Science.

[55] Dash S, Xiao C, Morgantini C, Koulajian K, Lewis GF (2015). Intranasal insulin suppresses endogenous glucose production in humans compared with placebo in the presence of similar venous insulin concentrations. Diabetes.

[56] Heni M (2017). Hypothalamic and Striatal Insulin Action Suppresses Endogenous Glucose Production and May Stimulate Glucose Uptake During Hyperinsulinemia in Lean but Not in Overweight Men. Diabetes.

[57] Sanguinetti E (2016). Maternal high-fat feeding leads to alterations of brain glucose metabolism in the offspring: positron emission tomography study in a porcine model. Diabetologia.

[58] Dinan TG, Cryan JF (2017). Gut instincts: microbiota as a key regulator of brain development, ageing and neurodegeneration. J Physiol.

[59] Wang J (2016). The Effects of LW-AFC on Intestinal Microbiome in Senescence-Accelerated Mouse Prone 8 Strain, a Mouse Model of Alzheimer’s Disease. J Alzheimers Dis.

[60] Akbari E (2016). Effect of Probiotic Supplementation on Cognitive Function and Metabolic Status in Alzheimer’s Disease: A Randomized, Double-Blind and Controlled Trial. Front Aging Neurosci.

[61] Junges VM, Closs VE, Nogueira GM, Gottlieb MGV (2018). Crosstalk between Gut Microbiota and Central Nervous System: A Focus on Alzheimer’s Disease. Curr Alzheimer Res.

[62] Matsueda S, Niiyama Y (1982). The effects of excess amino acids on maintenance of pregnancy and fetal growth in rats. J Nutr Sci Vitaminol (Tokyo).

[63] Dambacher S, Hahn M, Schotta G (2010). Epigenetic regulation of development by histone lysine methylation. Heredity (Edinb).

[64] Civitelli R (1992). Dietary L-lysine and calcium metabolism in humans. Nutrition.

[65] Vaz FM, Wanders RJ (2002). Carnitine biosynthesis in mammals. Biochem J.

[66] Shimazu S, Miklya I (2004). Pharmacological studies with endogenous enhancer substances: beta-phenylethylamine, tryptamine, and their synthetic derivatives. Prog Neuropsychopharmacol Biol Psychiatry.

[67] Ueda N, Tsuboi K, Uyama T (2013). Metabolism of endocannabinoids and related N-acylethanolamines: canonical and alternative pathways. FEBS J.

[68] Rapoport SI (2008). Arachidonic acid and the brain. J Nutr.

[69] Fernstrom John D., Fernstrom Madelyn H. (2007). Tyrosine, Phenylalanine, and Catecholamine Synthesis and Function in the Brain. The Journal of Nutrition.

[70] Lewerenz J, Maher P (2015). Chronic Glutamate Toxicity in Neurodegenerative Diseases-What is the Evidence?. Front Neurosci.

[71] Barrett E, Ross RP, O’Toole PW, Fitzgerald GF, Stanton C (2012). gamma-Aminobutyric acid production by culturable bacteria from the human intestine. J Appl Microbiol.

[72] Boix-Amoros A, Collado MC, Mira A (2016). Relationship between Milk Microbiota, Bacterial Load, Macronutrients, and Human Cells during Lactation. Front Microbiol.

[73] Schmieder R, Edwards R (2011). Quality control and preprocessing of metagenomic datasets. Bioinformatics.

[74] Caporaso JG (2010). QIIME allows analysis of high-throughput community sequencing data. Nat Methods.

[75] Segata N (2011). Metagenomic biomarker discovery and explanation. Genome Biol.

[76] Langille MG (2013). Predictive functional profiling of microbial communities using 16S rRNA marker gene sequences. Nat Biotechnol.

