# Supplementary material for: Microbiota signatures relating to reduced memory and exploratory behaviour in the offspring of overweight mothers in a murine model
Source: Sci Rep. 2019 Aug 30;9:12609. doi: 10.1038/s41598-019-48090-8 (PMC6717200; doi:10.1038/s41598-019-48090-8)
Supplement: Supplementary file 1 — Supplementary information [file 41598_2019_48090_MOESM1_ESM.pdf]

Microbiota signatures relating to reduced memory and exploratory behaviour in the offspring of overweight mothers in a murine model

Elena Sanguinetti[1,2*], Maria Angela Guzzardi[1*], Maria Tripodi[1], Daniele Panetta[1], Marta Selma-Royo[3], Alessandro Zega[1], Mauro Telleschi[1], Maria Carmen Collado[3#], Patricia Iozzo[1#]

*Equal contribution to first authorship

[#]Equal contribution to last authorship

[1]Institute of Clinical Physiology, National Research Council (CNR), via Moruzzi, 56124 Pisa, Italy

[2]Sant'Anna School of Advanced Studies, Pisa, Piazza Santa Caterina, 56100, Pisa, Italy

[3]Institute of Agrochemistry and Food Technology-National Research Council (IATA-CSIC), 46980, Valencia, Spain

Table S1: Multivariate canonical correspondence analysis (CCA) describing the correlations (p-values reported) between maternal habitus and gut microbiota composition

	Phylum	Class	Order	Family	Genus	OTU
**Age: 1 month**						
**Ceacum**	0.597	0.575	0.756	0.736	0.608	0.465
**Colon**	0.322	0.212	0.241	0.096	**0.041**	**0.021**
**Age: 6 months**						
**Caecum**	**0.016**	**0.034**	**0.013**	**0.016**	**0.02**	**0.015**
**Colon**	**0.014**	**0.017**	**0.014**	**0.011**	**0.004**	**0.002**

Statistically significant p-values are bolded

Table S2: Correlations between colon microbiota taxa, body weight and food intake.

	Bacteria Level	Bacteria taxa	R	p	FDR
**Weight at weaning**					
	Phylum	Bacteroidetes	0.47	0.02320	0.16
	Class	Bacteroidia	0.47	0.02320	0.28
	Order	Bacteroidales	0.47	0.02320	0.37
	Family	Clostridiaceae	-0.6	0.00239	0.067
	Family	S247	0.48	0.02068	0.17
*	Family	Peptostreptococcaceae	-0.47	0.02437	0.17
*	Family	Rikenellaceae	-0.47	0.02490	0.17
	Genus	Unclassified.Clostridiaceae	-0.6	0.00225	0.088
	Genus	Unclassified.S247	0.48	0.02068	0.24
*	Genus	Unclassified.Peptostreptococcaceae	-0.47	0.02437	0.24
*	Genus	Unclassified.Rikenellaceae	-0.47	0.02490	0.24
	Genus	Unclassified	0.41	0.04984	0.39
**Weight at 6 months**					
	Phylum	Unclassified	0.59	0.01054	0.074
	Class	Unclassified	0.59	0.01007	0.12
	Order	Unclassified	0.59	0.01007	0.17
	Family	Peptostreptococcaceae	-0.62	0.00614	0.2
*	Family	Rikenellaceae	0.49	0.03710	0.38
	Family	Unclassified	0.47	0.04879	0.38
*	Family	Porphyromonadaceae	0.45	0.06040	0.38
	Genus	Unclassified.Peptostreptococcaceae	-0.62	0.00614	0.29
	Genus	Bilophila	0.52	0.02744	0.49
*	Genus	Unclassified.Rikenellaceae	0.49	0.03710	0.49
*	Genus	Parabacteroides	0.45	0.06040	0.49
**Daily food intake at 6 months**					
	**Phylum**	**Unclassified**	**0.63**	**0.00490**	**0.034**
*	Phylum	Proteobacteria	0.53	0.02221	0.078
	**Class**	**Unclassified**	**0.64**	**0.00452**	**0.031**
*	**Class**	**Deltaproteobacteria**	**0.63**	**0.00517**	**0.031**
	**Order**	**Unclassified**	**0.64**	**0.00452**	**0.044**
*	**Order**	**Desulfovibrionales**	**0.63**	**0.00517**	**0.044**
*	Order	Bifidobacteriales	0.57	0.01389	0.079
*	Order	Actinomycetales	-0.49	0.04067	0.17
*	Family	Desulfovibrionaceae	0.63	0.00517	0.068
*	Family	Rikenellaceae	0.61	0.00703	0.068
	Family	Peptostreptococcaceae	-0.61	0.00724	0.068
	Family	Unclassified	0.6	0.00849	0.068
*	Family	Bifidobacteriaceae	0.57	0.01389	0.089
*	Genus	Unclassified.Rikenellaceae	0.61	0.00703	0.17
	Genus	Unclassified.Peptostreptococcaceae	-0.61	0.00724	0.17
	Genus	Bifidobacterium	0.57	0.01389	0.17
	Genus	Unclassified	0.57	0.01419	0.17
*	Genus	Desulfovibrio	0.55	0.01747	0.17
	Genus	Adlercreutzia	0.48	0.04237	0.34

Correlations remaining statistically significant after FDR correction are in bold. Bacterial taxa associated with at least one of the cognitive parameters (alternation triplets, arm entries, distance) and/or maternal habitus are indicated with an asterisk (*)

Table S3. Multivariate canonical correspondence analysis (CCA) describing the correlations (p-values reported) between maternal habitus and expression of KEGG pathways inferred from gut microbiota

	**p-value**
**Age: 1 month**	
Caecum	0.658
Colon	0.292
**Age: 6 months**	
Caecum	0.115
Colon	**0.050**

Statistically significant p-values are bolded

Figure S1 Bacterial taxa at OTU level identified by LEfSE analysis

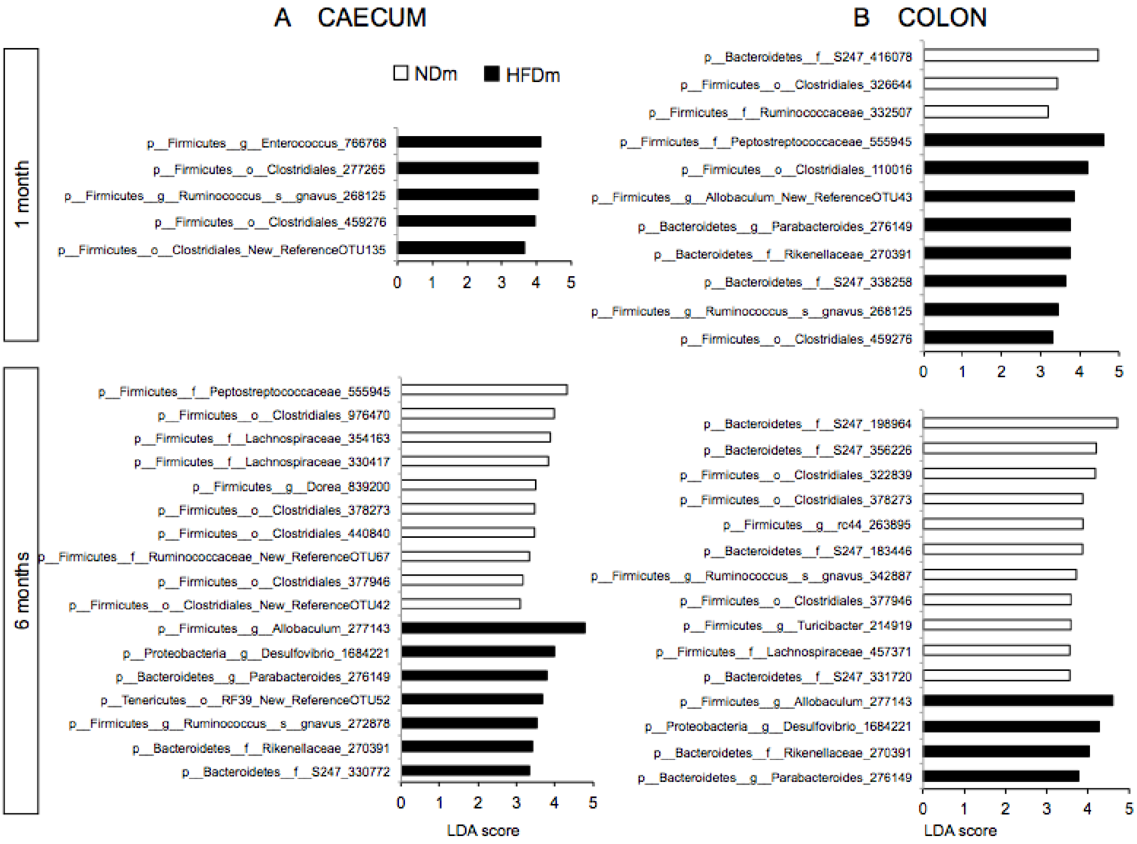

Fig. S1. Bacterial taxa at OTU level identified by LEfSE anaylsis (LDA score>3), and explaining the difference in microbiota composition in the caecum (A) and in the colon (B) between offspring of mothers fed normal diet (NDm) or high-fat diet (HFDm) at 1 (top panel) and 6 months of age (bottom panel).

Figure S2. Colon microbiota-derived Kyoto Encyclopaedia of Genes and Genomes (KEGG)

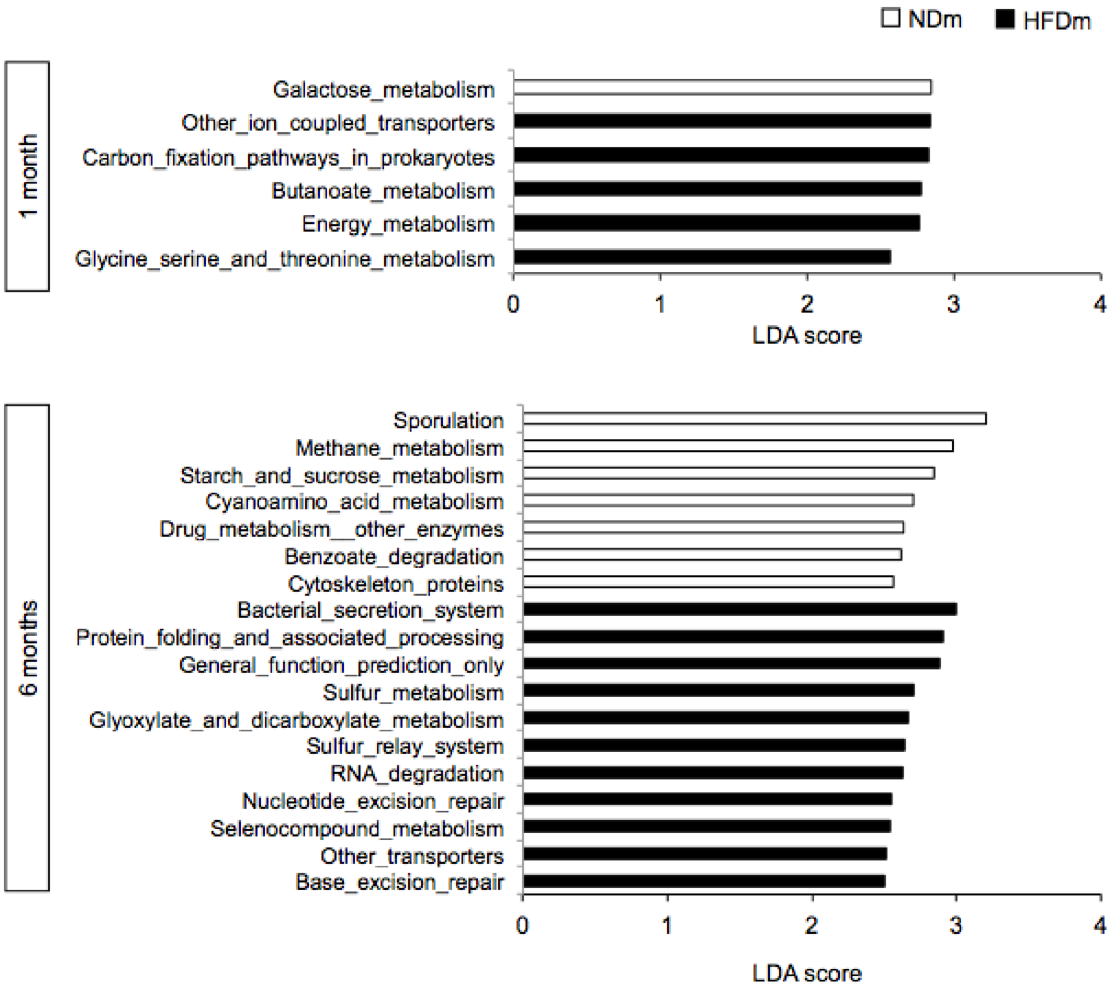

Fig. S2. Colon microbiota-derived Kyoto Encyclopaedia of Genes and Genomes (KEGG) pathways explaining functional microbiota differences between offspring of mothers fed normal diet (NDm) or high-fat diet (HFDm) at 1 (top panel) and 6 months of age (bottom panel).
